# Comparative Proteomics Analyses Reveal the *vir*B of *B. melitensis* Affects Expression of Intracellular Survival Related Proteins

**DOI:** 10.1371/journal.pone.0005368

**Published:** 2009-04-29

**Authors:** Yufei Wang, Zeliang Chen, Feng Qiao, Tianyi Ying, Jing Yuan, Zhijun Zhong, Lei Zhou, Xinying Du, Zhoujia Wang, Jin Zhao, Shicun Dong, Leili Jia, Xitong Yuan, Ruifu Yang, Yansong Sun, Liuyu Huang

**Affiliations:** 1 Institute of Disease Control and Prevention, Academy of Military Medical Science, Beijing, China; 2 Institute of Microbiology and Epidemiology, Academy of Military Medical Science, Beijing, China; 3 Beijing Institute of Pharmaceutical Chemistry, Beijing, China; University of California Merced, United States of America

## Abstract

**Backgound:**

*Brucella melitensis* is a facultative, intracellular, pathogenic bacterium that replicates within macrophages. The type IV secretion system encoded by the *virB* operon (*virB*) is involved in *Brucella* intracellular survival. However, the underlying molecular mechanisms, especially the target proteins affected by the *virB*, remain largely unclear.

**Methodology/Principal Findings:**

In order to define the proteins affected by *virB*, the proteomes of wild-type and the *virB* mutant were compared under in vitro conditions where *virB* was highly activated. The differentially expressed proteins were identified by MALDI-TOF-MS. Forty-four down-regulated and eighteen up-regulated proteins which exhibited a 2-fold or greater change were identified. These proteins included those involved in amino acid transport and metabolism, lipid metabolism, energy production, cell membrane biogenesis, translation, post-translational modifications and protein turnover, as well as unknown proteins. Interestingly, several important virulence related proteins involved in intracellular survival, including VjbR, DnaK, HtrA, Omp25, and GntR, were down-regulated in the *virB* mutant. Transcription analysis of virB and vjbR at different growth phase showed that virB positively affect transcription of vjbR in a growth phase dependent manner. Quantitative RT-PCR showed that transcription of these genes was also affected by *virB* during macrophage cell infection, consistent with the observed decreased survival of the *virB* mutant in macrophage.

**Conclusions/Significance:**

These data indicated that the *virB* operon may control the intracellular survival of *Brucella* by affecting the expression of relevant proteins.

## Introduction

Brucellosis, also known as undulant or Malta fever, is one of the most common bacterial zoonoses endemic in many countries, particularly developing ones [1]. Brucellosis is caused by the genus *Brucella*, which consists of seven species according to antigenic variation and primary host. In general, humans can be infected by *B. melitensis*, *B. abortus*, and *B. suis*. The pathological manifestations of *Brucellosis* in humans include meningitis, endocarditis, spondylitis, and arthritis. *Brucella* infection occurs through inhalation or ingestion of the organisms. Following penetration of the epithelium, the bacteria are transported, either free or within phagocytes, to the regional lymph nodes and then to different tissues [2]. *Brucella* species can survive within professional and non-professional phagocytes. Mutant strains that lose intracellular survival cannot carry out infection of their host; therefore, the virulence of *Brucella* depends upon its ability to survive and replicate within host cells.

For successful intracellular survival, an invading bacterial pathogen must overcome the bactericidal mechanisms employed by its host [3]. Intracellular bacterial pathogens have developed various ways to circumvent host defense or bacterial degradation, such as controlling the maturation of their hosts' membrane-bound compartments and transforming them into nutrient-rich environments where they can replicate [4], [5], [6], [7]. Following internalization, *Brucella* redirects the vacuolar traffic in a way that avoids endocytosis and inhibits phagosome-lysosome fusion [4]. *Brucella* transits through the cell via the autophagosome-like *Brucella*-containing vacuole (BCV). Subsequent interactions of this BCV with the ER membrane allow its maturation into an intracellular replication compartment where the bacteria can multiply. *Brucella* displays unique virulence characteristics, as many typical virulence determinants, including type I, II, and III secretion systems and pathogenicity islands, are absent from this bacteria. Therefore, the underlying virulence mechanisms employed by *Brucella* remain largely unknown. In a mutant screen for *B. suis* virulence factors, the *virB* operon (*virB*) consisting of 11 open-reading frames was found to be essential for *B. suis* persistence in mice [8]. *VirB* shares homology with other bacterial type IV secretion systems involved in intracellular trafficking and survival. *VirB* is also induced in macrophages, where it is required for localization of *Brucella* to mature BCV [4].

Biochemical and pathological studies further define the roles of *virB* in *Brucella* survival. *Brucella* invasion induces acidification of the intracellular environment, which can enhance *virB* expression [9]. Furthermore, *virB* mediates BCV maturation [10], and is essential for bacterial survival in both the early and late stages of infection. In early infection, BCV of *virB* mutant but not wild type *Brucella* fuse with host cell lysosomes, resulting in degradation of the mutant but not the wild type pathogen. In the late stages of infection, *virB* is indispensable for sustained interactions between *Brucella* and the ER, and hence BCV maturation [4]. It is also important to note that *virB* is tightly regulated throughout this process of host invasion and bacterial replication. As is the case for other secretion system, *virB* is tightly regulated by other signals, and its main functions are carried out by the effectors molecules. These molecules regulate expression of other genes or interact with host cell. C Nijskens et al proved that the effectors secreted by a WT strain could rescue the trafficking deficiency of a ΔvirB mutant during co-infection in cells [11]. Taken together, these results suggest that *virB* mediates *Brucella* survival by affect expression of other genes and modifying signaling pathways of host cells. The identification of these affected genes will provide more information about the function and virulence roles of *virB*.

Our goal of the present study was to identify the genes affected by *virB*, thereby explaining the molecular mechanisms underlying *Brucella* virulence and the role of *virB*. A comparative proteome approach was used to identify the differentially expressed proteins affected by *virB*. Several of differentially expressed proteins were selected for transcription during macrophage infection. The results indicated that *virB* affect the expression of a number of other proteins, which may be involved in *Brucella* intracellular survival.

## Materials and Methods

### Construction and complementation of the virB mutant BMΔvirB

A *virB* inactivation mutant BMΔvirB (BM with promoter of the *virB* operon deleted) and complementary strains BM-IVGT (BMΔvirB containing complementary plasmid pBBR1-IVGT) were constructed from *B. melitensis* 55009, a strain derived from 16 M. The 1.7 kb *SacB* was released from pKOBEG-*SacB* by NdeI and cloned into the *Nde*I site of pUC19 to give pUC19-*SacB*. This allowed positive selection of double cross-over events using sucrose resistance as selection. A 504 bp region just upstream of *vir*B1 was deleted as follows: The N-terminal fragment was amplified with primers IVB-N-F and IVB-N-R, and the C-terminal fragment with IVB-C-F and IVB-C-R. The two PCR products were purified, equally mixed, and then amplified with IVB-N-F and IVB-C-R to generate IVB that was cloned into pUC19-*SacB* to generate pUC*SacB*-IVB. BM was electroporated with pUC*SacB*-IVB and amp^S^ sucrose^R^ colonies were isolated. The unmarked deletion mutant was further confirmed by PCR and DNA sequencing.

The complementary strain of BMΔvirB was constructed as follows: A 1087 bp fragment located downstream of the *vir*B operon was amplified with IVGT-F and IVGT-R and cloned into pUC19 to generate the targeted plasmid pUC19-IVT. This plasmid was transformed into competent BM, resulting in the targeted strain BM-IVT. Genomic DNA was extracted from BM-IVT and digested with *Eco*T22I, and then purified, self-ligated, and transformed into DH5α to rescue the plasmid pUC19-IVGT. The rescued *vir*B operon in pUC19-IVGT was confirmed by PCR with primers for *vir*B1, *vir*B2, *vir*B5, and *vir*B8. PCR products were sequenced to confirm the sequence. The two termini of the rescued fragment were confirmed by sequencing with sequencing primers of pUC19. The *vir*B operon was released from pUC19 by digestion with *Kpn*I and *Pst*I and then subcloned into pBBR1MCS-5 to generate pBBR1-IVGT. The plasmid pBBR1-IVGT was electroporated into the BM**Δ**virB mutant, resulting in the complementary strain BM-IVGT. The complementary strain was confirmed by semi-quantitative RT-PCR for *vir*B1 and *vir*B8.

### Determination of in vitro induction conditions for virB

*Brucella* was grown in TSB to the logarithm phase (*OD*_600_ = 1.0) at 37°C and then subjected to different stress conditions modified from previous study [12]. The bacteria were subjected to TSB4.0 (acid shock), TSB5.5 (acid shock), GEM7.0 (MgSO_4_.7H_2_O 0.2 g/L,Citric acid. H_2_O 2.0 g/L,K_2_HPO_4_ 10.0 g/L,NaNH_4_HPO_4_.4H_2_O 3.5 g/L,Glucose 20 g/L, pH 7.0 , limited nutrition) [13] , GEM4.0 (limited nutrition and acid shock), TSB with 1.5 mM H_2_O_2_ (oxidative stress), TSB with 50 mM H_2_O_2_ (oxidative stress), 42°C (heat shock), TSB7.0 (control) for 30 min. Then, the transcription of *virB* under these stresses was compared by quantitative RT-PCR.

### Preparation of the whole cell protein extract

The BM and BMΔvirB strains were grown in TSB at 37°C to the middle logarithmic phase (*OD_600_* = 1.0), and then transferred to the stress condition where *virB* was greatly activated. Bacterial cells were harvested by centrifugation and cell pellets were re-suspended in 5 ml of lysis buffer (7 M urea, 2 M thiourea, 4% (w/v) CHAPS, and 50 mM DTT) containing complete protease inhibitors (Roche Applied Science, Indianapolis, IN). The cells were sonicated for 10 min on ice using a Sonifier 750 (Branson Ultrasonics Corp., Danbury, CT) with the following parameters: 2 s of sonication with a 2 s interval, 35% duty cycle. After addition of 2.5 mg of RNase (Promega, Madison, WI) and 100 units of DNase (Promega, Madison, WI), the cell lysate was incubated for 1 h at 15°C to solubilize proteins. The lysate was then centrifuged at 20,000 g for 20 min to pellet the insoluble components. The supernatant was collected, and protein concentration was measured using the PlusOne 2-D Quant kit (Amersham Pharmacia Biotech, Sweden). 800 µg aliquots of the purified proteins were stored at −70°C.

### Two-dimensional Polyacrylamide Gel Electrophoresis

Two-dimensional polyacrylamide gel electrophoresis (2-DE) was performed as follows: 18 cm IPG strips (pH ranges, 4–7) (Amersham Pharmacia Biotech) were used for isoelectric focusing (IEF). 800 µg of total protein extract was loaded and IEF was conducted at 20°C for 50,000 Vhrs. The maximum voltage and current were set as 8,000 V and 50 µA/strip, respectively. After IEF, each strip was equilibrated in 10 ml equilibration buffer 1 (6 M urea, 0.5% DTT, 30% glycerol, 50 mM Tris-Cl pH 8.8) for 15 min, and then in 10 ml of equilibration buffer 2 (6 M urea, 4.5% iodoacetamide, 30% glycerol, 50 mM Tris-Cl pH 8.8) for another 15 min. The vertical slab SDS–PAGE (12.5%) was run with 30 mA/gel in a ProteanTM II XL system (Bio-Rad, Hercules, CA). Preparative gels used for identification of proteins by mass spectrometry were stained with Coomassie Brilliant Blue R-350 (Ameresco Co., Solon, OH, USA). Gels were scanned and images were analyzed with ImageMaster™ 2D Platinum software (Amersham Biosciences, Uppsala, Sweden). The relative volume of each spot was determined from the spot intensities in pixel units and normalized to the sum of the intensities of all the spots of the gel. Proteins with at least 2-fold volume variations were considered to be differentially expressed.

### In-gel protein digestion

The protein spots of interest were cut out of the gel and de-stained with 50 µl of 25 mM ammonium bicarbonate in 50% acetonitrile (ACN) for 30 min at room temperature for three times. The de-stained gel pieces were completely dried in a SpeedVac vacuum concentrator (Savant Instruments, Farmingdale, NY, USA). The gels were re-swollen with 3 µl of 25 mM ammonium bicarbonate containing 10 µg of trypsin at 4°C for 1 h. After 12 h of incubation at 37°C, the gels were dried under high vacuum centrifuge to evaporate the solvent. 8 µl of 5% trifluoroacetic acid (TFA) was added to the gel spots and incubated at 37°C for 1 h. The supernatant was transferred into a new microtube. 8 µl of 2.5% TFA in 50% ACN was added and incubated at 30°C for 1 h. The supernatant was transferred to a new microtube. At last 8 µl 100% ACN was used for extraction of hydrophobic peptides. All of the supernatants were combined and dried in the SpeedVac vacuum concentrator, and re-solubilized with 3 µl of 0.5% TFA in 30% ACN.

### MALDI-TOF-MS

All matrix-assisted laser desorption/ionization time-of-flight mass spectroscopy (MALDI-TOF-MS) measurements were performed on a Bruker Reflex III MALDI-TOF-MS (Bruker Daltonik, Bremen, Germany) operating in reflectron mode. A saturated solution of CHCA in 50% ACN and 0.1% TFA was used as the matrix solution. One microliter of the matrix solution and sample solution with a 1∶1 ratio were mixed and applied onto the Score384 target well. The MALDI-TOF-MS analysis was performed at 20 kV accelerating voltage and 23 kV reflecting voltage.

### Peptide mass fingerprinting

Peptide mass fingerprinting (PMF) searches were performed by using the program MASCOT developed by Matrix Science (http://www.matrixscience.com). For protein identification, peptide masses searches against the NCBInr database with free access on the Internet were done. Monoisotopic peptide masses were used to search the databases, allowing a peptide mass accuracy of 100 ppm and one partial cleavage. The search parameters were: maximum of one missed cleavage by trypsin; fixed modification of oxidized methionine. Using these parameters and searching only the *B. melitensis* 16 M database, probability based MOWSE scores greater than 48 are significant (p<0.05). For unambiguous identification of proteins more than five peptides must be matched and the sequence coverage must be greater than 15%.

### RNA Sample Preparation and cDNA Synthesis

For transcription analysis of *virB* under in vitro conditions, bacteria were cultured in TSB to logarithmic phases (*OD_600_* = 1.0) and then subjected under specific conditions. Total RNA were isolated with Trizol agent (Invitrogen, Carlsbad, CA) as recommended by the manufacturer. RNA samples were then treated with DNAse I (Promega, Madison, WI) to remove any contaminating genomic DNA. RNA quantity and quality was assessed using ND-1000 Spectrophotometer Nanodrop (Technologies, CA). Complementary DNA (cDNA) was generated from total RNA using a random hexamer primer following the protocol for Superscript II (Invitrogen, Carlsbad, CA).

### Semi-quantitative RT-PCR

For simi-quantitative RT-PCR analysis, 16S rRNA, whose transcription is relatively constant in bacterial, was chosen as internal control. Different cDNA samples were amplified with primers for 16S rRNA, and the cDNA samples were normalized by differential dilutions according to quantity of 16S rRNA products. Then, selected genes were amplified from normalized cDNA samples with specific primers (The primers used for RT-PCR assays were listed in Table 1). The PCR products were analyzed on 1.2% agarose gel and visualized by ethidium bromide staining.

**Table 1:** Primers used in this study.

Primers	Sequences (5′–3′)
IVB-N-F	CTGCGAAGCTTGCAAATTCCCGTCCGGTTCG
IVB-N-R	GAGGACAAGGAATGGCACCACGACGCAGGACGGAAAGGAC
IVB-C-F	GTCCTTTCCGTCCTGCGTCGTGGTGCCATTCCTTGTCCTC
IVB-C-R	CGACCGGAATTCGAAGCCGCCCGTAAAGTTGC
IVGT-F	ACGTCGGATCCGAATTCACAGGCATTATCCGCTCGTC
IVGT-R	AGTCGAAGCTTTCTAGAAGCATAGCCAGTAGGTCCAG
PRO-F	GAGCGGCTGGAACTGCAAAC
PRO-R	GACCAACCGCCCACCAACGAC
virB1-RT-F	AAGCAATCACGACAGCACAG
virB1-RT-R	CGGCGTAGTAACAGGAGAATG
virB8-RT-F	GGGCTTTCGGCACCATTAC
virB8-RT-R	AGCGTGTACCAGTCGTAGG
16sRNA-RT-F	CACTGGACCATTACTGACGC
16sRNA-RT-R	ACTAAGGGCGAGGGTTGC
dnaK-RT-F	TGAAATGGCAGCCGATAA
dnaK-RT-R	AAGCGAGGTCTTGAGGG
gntR-RT-F	AAAATGACCGAAGCATCTGG
gntR-RT-R	TGCGGGAAATGGGACGAA
htrA-RT-F	TTTGGCGACGATAATAAGGTG
htrA-RT-R	ATGGCGAGAAGATGGCG
omp25-RT-F	CAGCACCGTTGGCAGCAT
omp25-RT-R	GGCATAACCGGGTTCAGG
vjbR-RT-F	CGAGGTGGAGGACGAAGA
vjbR-RT-R	ATAATGCCGAGGGAAAGC

### Real-Time Quantitative RT-PCR (qRT-PCR)

Samples were run in triplicate and amplified in a 20 µl reaction containing 10 µl of 2×SYBR Green I Master Mix (TAKARA, Japan), 100 nM of forward/reverse primer, and 1 µl of cDNA target. Moreover, no-template controls were used as recommended. The mix was incubated at 95°C for 15 s, and then cycled at 95°C for 5 s and at 60°C for 20 s 50 times using the Roche LightCycler2.0 system. Relative transcriptional level was determined by the methods of 2^−ΔΔCt^ as described previously [14]. Relative fold change (Treatment/control) = 2^−ΔΔCt^, where ΔCt (Gene of interest) = Ct (Gene of interest)-Ct (Reference gene of the same sample) and ΔΔCt (Gene of interest) = ΔCt (Treatment)−ΔCt (Control). The 16S rRNA, the expression of which is relatively constant in bacteria, was used as a reference gene and bacterial grown in TSB7.0 was used as the control.

### Macrophage infection assay, growth curve and transcription analysis

Macrophage cell infection was assayed as described earlier [15]. Murine macrophage-like J774A.1 cells were seeded in 24-well plates at 5×10^5^ cells/well. Cells were incubated in 5% CO_2_ at 37°C for 8 h and then infected with *Brucella* at an MOI of 200. To synchronize the infection, the infected plates were centrifuged at 200 g for 5 min at room temperature. Following 60 min incubation, the cells were washed three times with PBS to remove extracellular bacteria. To assess intracellular growth of the bacterial, the cell were incubated for 1 h in DMEM/F12 supplemented with 100 µg/ml of ampicilin plus 50 µg/ml of kanamycin to kill extracellular bacteria, then the concentration of antibiotics were reduced to 20 µg/ml (time zero). At different time points post infection, the supernatant was discarded and cells were lysed with 0.1% (v/v) Triton X-100. The CFU were obtained by plating serial dilutions on TSA plates. All the infections were performed in triplicate and repeated for 3 times. For transcription analysis during macrophage infection, at different time points post infection, the total RNA was isolated with Trizol agent. The reverse transcription and qRT-PCR analysis was carried out as described above. RNA of J774A.1 uninfected was isolated as a negative control. For growth curve analysis, bacteria were grown in TSB for 24 h, and then diluted with TSB to OD_600_ = 0.05 and cultured at 37°C with shaking. Aliquots of the cultures were taken at an interval of 2 h and cell density OD_600_ was recoded. Transcription of *vir*B and *vjb*R at different growth stages were analyzed by qRT-PCR.

## Results

### Construction and confirmation of the virB mutant and complementary strains

The 11 sequential ORFs encoding Type IV secretion machinery comprise *vir*B, making *vir*B inactivation possible by promoter deletion. To construct an unmarked deletion mutant of *vir*B, the counter-selection gene *Sac*B was firstly cloned into pUC19 to give pUC19-*SacB*. This plasmid was then used to construct the *vir*B mutant as described above. The unmarked deletion mutant BMΔvirB, which resulted from two rounds of croos-over, was obtained by its amp^S^ and sucrose^R^ phenotypes (data not shown).

**Figure 1:**
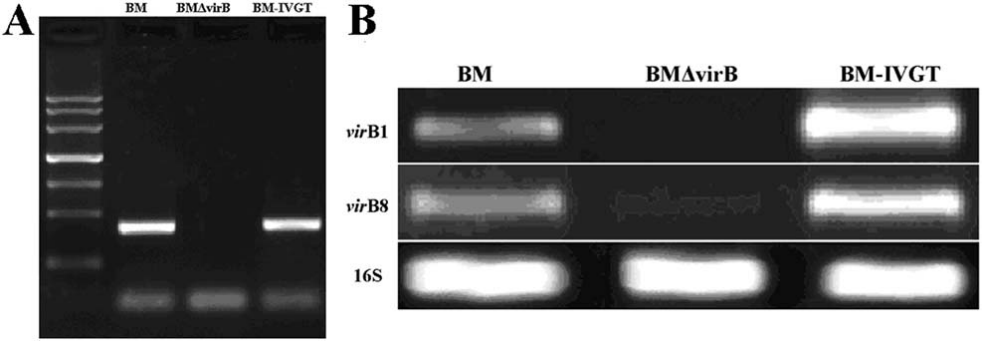
Construction and confirmation of BMΔvirB and BM-IVGT strains. A) The promoter was amplified from BM and BM-IVGT, but not from BMΔvirB, showing that the promoter was deleted in BMΔvirB and complemented in BM-IVGT. B) RT-PCR amplification of *vir*B1 and *vir*B8 showed that the two genes could be amplified from BM and BM-IVGT, but not BMΔvirB, indicating that transcription of *vir*B genes was inactivated in BMΔvirB and restored in BM-IVGT.

To construct the complementary strain, *vir*B was cloned by plasmid rescue and sub-cloned into pBBR1MCS5, a plasmid that could replicate in *Brucella* [16], [17], to yield complementary plasmid pBBR-IVGT. PCR verification and DNA sequencing showed that the virB operon was correctly rescued and cloned in pBBR-IVGT. This plasmid was transformed into BMΔvirB, generating the complementary strain BM-IVGT. PCR verification demonstrated that the promoter region was deleted in BMΔvirB and recovered in BM-IVGT (Figure 1A). Semi-quantitative RT-PCR indicated that transcription of the *vir*B genes *vir*B1 and *vir*B8 was detected in BM and BM-IVGT, but not BMΔvirB, indicating that *vir*B was successfully inactivated in BMΔvirB and restored in BM-IVGT (Figure 1B). Interestingly, transcription of *vir*B genes in BM-IVGT was higher than that in BM (Figure 1B). pBBR1MCS5 is a wide host range plasmid that could replicate in *Brucella* in multi-copy[16], [17]. This might be the reason for the increased transcription of *vir*B genes in BM-IVGT compared to BM.

### Determination of in vitro induction conditions for virB

**Figure 2:**
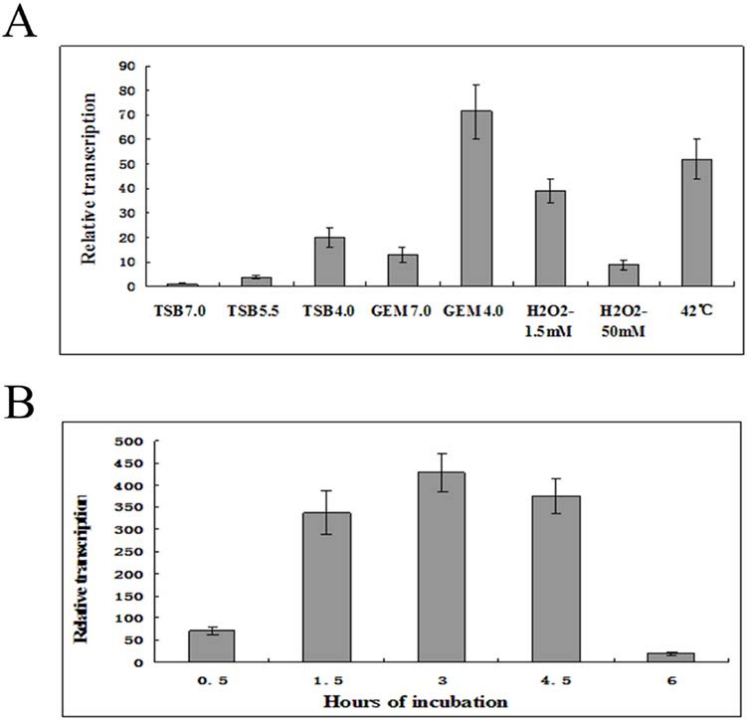
Determination of in vitro induction conditions of *virB*. A: Transcription of *virB* under different in vitro condition. BM was firstly cultured in TSB to logarithmic phase and then subjected to different stresses. RNA was isolated and transcription of *virB* was quantified by qRT-PCR. *virB* was greatly activated under GEM4.0. B: Transcription of *virB* at different incubation time in GEM4.0. BM was subjected to different incubation time in GEM4.0 and then transcription of *virB* was quantified. The *virB* was greatly activated at 3 h.

Although *virB* is mainly activated during host infection, it is also activated under in vitro conditions [9]. Due to the difficulty in isolation of enough bacteria proteins from infection mixtures and the contamination of host cell proteins, we chose to compare proteomes of BM and BMΔvirB cultured in vitro. To greatly differentiate protein expressions between BM and BMΔvirB, we sought to compare their proteomes under in vitro conditions where *virB* was highly activated. To identify such conditions, BM was subjected to several in vitro stress treatments as described previously, and the relative transcription of the *virB* was quantified. *virB* was greatly activated in acidified minimum medium (GEM 4.0) (Figure 2A). To ensure that nutrition limitation did not inhibit growth of *Brucella*, the incubation time in GEM 4.0 was determined. Transcription of *virB* peaked at 3 h (Figure 2B). Therefore, 3 h of incubation in GEM 4.0 was used in comparative proteomic analyses.

### Overview of the comparative proteome

*Brucella* was firstly cultured in TSB to logarithmic phase, and then transferred to acidified minimum for 3 h of incubation. Whole cell proteins were extracted, followed by isoelectric focusing and SDS-PAGE. To obtain an overview of the protein distribution, we used pH 3–10 IPG strips (180 mm) first (data not shown). The result shows that most proteins' pI is located between 4 and 7, so finally we chose the strips with a range of pH 4–7.

**Figure 3:**
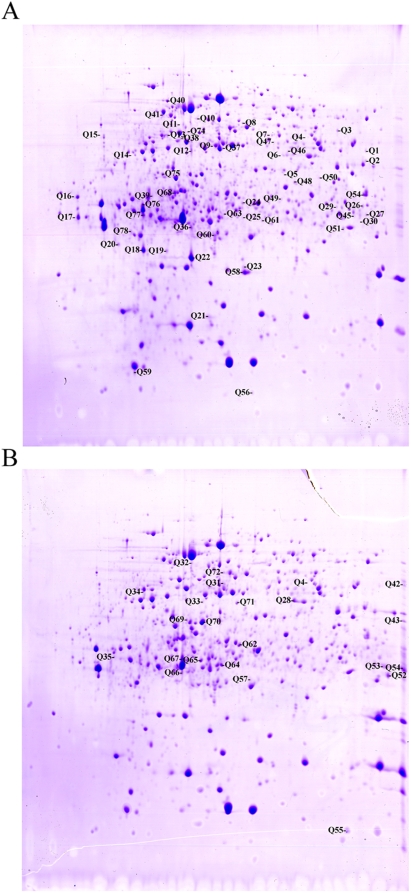
Proteomes of *B. melitensis* strains BM and BMΔvirB in the pH range of 4.0 to 7.0. BM (A) and BMΔvirB (B) were firstly cultured in TSB to logarithmic phase and then transferred into GEM4.0 for 3 h. Protein extracts (800 µg) of each strain were focused with IPG strips and run on 12% SDS-PAGE gels. The gels were stained with Coomassie Brilliant Blue R-350 and subjected to 2 DE analyses. The gels of BM and BMΔvirB were scanned and compared with ImageMaster™ 2D Platinum software. The labeled protein spots were the ones whose expressions were changed over 2 folds.

**Figure 4:**
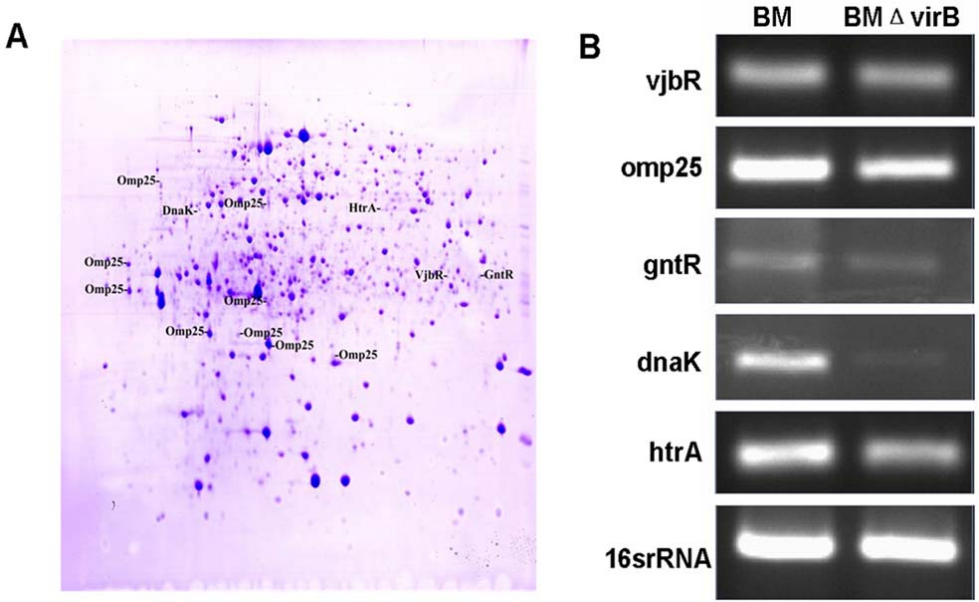
Confirmation of comparative proteome by semi-quantitative RT-PCR. A: Spot distribution of the selected virulence related protein on gel of BM. Several protein spots with different MW and pI of *omp25* were identified. B: Relative transcription of virulence related genes in BM and BMΔvirB. BM and BMΔvirB were firstly cultured in TSB to logarithmic phase and then transferred into GEM4.0 for 3 h. RNA was isolated and relative transcription of virulence related genes was quantified by normalization with 16S rRNA. These genes were transcribed at a lower level in BMΔvirB than in BM.

The typical proteome gel maps of BM and BMΔvirB are presented in Figure 3. A total of 951 and 964 protein spots were detected for strains BM and BMΔvirB, respectively. 910 protein spots of the two strains could be matched. The abundance of a protein is calculated as the Vol% which represents the ratio of it to all the detected protein spots. The relative expression change is the abundance ratio of a spot matched between the two strains. According to this criteria, those whose abundance changed 2 fold or greater were considered as greatly differentially expressed proteins. When compared with BM, 59 protein spots were down-regulated, and 36 were up-regulated in BMΔvirB. 19 protein spots were uniquely expressed in BM, and 27 in BMΔvirB (data not shown). These protein spots were cut out from the 2-DE gel and subjected to in gel digestion, followed by MALDI-TOF-MS identification. Peptide mass fingerprinting were searched with MASCOT for protein identification. Only those whose peptide covered more than 15% of the amino acid sequence were considered as positive identification. 76 protein spots representing products of 62 proteins were successfully identified. Of the 62 proteins, 44 proteins were downshifted or repressed in BMΔvirB, and 18 proteins were upshifted or induced (Table 2). These differentially expressed proteins are mainly involved in amino acid transport and metabolism (10/62), lipid metabolism (4/62), energy production (6/62), cell membrane biogenesis (6/62), translation (5/62), post-translational modifications and protein turnover (5/62), as well as unknown proteins. Among the identified proteins, 34 were cytoplasmic proteins. Interestingly, several known virulence related proteins, including VjbR, HtrA, GntR, Omp25 and DnaK, were down-regulated when *virB* was inactivatived (Figure 4A).

**Table 2:** List of differentially expressed proteins identified by MALDI-TOF-MS.

Spot no	NCBI GI	Locus	Gene	Protein description	Theoretical/Exptl		sequence coverage	COG^a^	Location^b^	Change fold^c^
					MW(kDa)	pI				
Energy production and conversion										
Q39	gi|17986535	BMEI0251	atpD	ATP synthase subunit B	55/34	5.48/4.95	0.51	C	U	Y
Q1	gi|17989212	BMEII0867	adh	alcohol dehydrogenase class III	40/41	6.03/6.63	0.29	C	C	−6.7
Q47	gi|17988739	BMEII0394	-	glycerol trinitrate reductase	40/43	5.80/5.91	0.35	C	C	−4.8
Q77	gi|17986535	BMEI0251	atpD	ATP synthase subunit B	55/31	5.48/4.88	0.31	C	U	−2.6
Q3	gi|17989214	BMEII0869	putA	succinate-semialdehyde dehydrogenase (NADP+)	52/46	6.42/6.42	0.41	C	C	−2.0
Q74	gi|17986422	BMEI0138	sucC	succinyl-CoA synthetase subunit beta	43/44	4.90/5.03	0.54	C	C	−2.0
Q4	gi|17988102	BMEI1819	adh	alcohol dehydrogenase class III	40/44	5.72/6.18	0.44	C	C	2.0
Amino acid transport and metabolism										
Q27	gi|17989216	BMEII0871	aroE	shikimate 5-dehydrogenase	30/31	5.99/6.64	0.27	E	U	−4.0
Q10	gi|17987926	BMEI1643	argE	N-carbamoyl-L-amino acid amidohydrolase	45/51	5.17/5.30	0.36	E	C	−2.9
Q51	gi|17989218	BMEII0873	livF	high-affinity branched-chain amino acid transport ATP-binding protein LivF	28/30	6.99/6.45	0.70	E	C	−2.7
Q49	gi|17987935	BMEI1652	ureC	urease subunit alpha	61/33	5.43/5.97	0.31	E	C	−2.6
Q7	gi|17988264	BMEI1981	-	aminopeptidase T	46/45	5.65/5.90	0.32	E	C	−2.5
Q50	gi|17987454	BMEI1171	argC	N-acetyl-gamma-glutamyl-phosphate reductase	34/37	5.89/6.27	0.54	E	C	−2.2
Q35	gi|17987387	BMEI1104	-	arginine/ornithine-binding periplasmic protein precursor	27/31	4.77/4.72	0.61	ET	P	T
Q53	gi|17988946	BMEII0601	-	cystine-binding periplasmic protein precursor	28/30	7.74/6.77	0.54	ET	P	9.3
Q65	gi|17988895	BMEII0550	proX	glycine betaine/l-proline-binding protein ProX	32/32	5.57/5.34	0.58	E	P	2.2
Q71	gi|17988978	BMEII0633	livK	leucine-, isoleucine-, valine-, threonine-, and alanine-binding protein precursor	39/40	5.52/5.62	0.56	E	P	2.2
Q54	gi|17988946	BMEII0601	-	cystine-binding periplasmic protein precursor	28/30	7.74/6.90	0.54	ET	P	2.1
Nucleotide transport and metabolism										
Q30	gi|17987061	BMEI0778	adk	adenylate kinase	21/31	6.63/6.59	0.57	F	C	−2.1
Carbohydrate transport and metabolism										
Q34	gi|17988062	BMEI1779	pfkB	Fructokinase	36/42	4.85/4.94	0.60	G	C	T
Q43	gi|17986593	BMEI0310	gapA	glyceraldehyde-3-phosphate dehydrogenase	36/38	6.13/6.91	0.71	G	C	2.7
Lipid transport and metabolism										
Q63	gi|17988859	BMEII0514	fabG	3-ketoacyl-(acyl-carrier-protein) reductase	27/31	5.44/5.52	0.59	IQR	C	−3.2
Q73	gi|17988206	BMEI1923	caiA	isovaleryl-CoA dehydrogenase	42/42	5.36/5.33	0.53	I	U	−2.2
Q44	gi|17986323	BMEI0039	accA	acetyl-CoA carboxylase carboxyltransferase subunit alpha	35/34	6.14/6.62	0.70	I	C	−2.1
Q9	gi|17988206	BMEI1923	caiA	isovaleryl CoA dehydrogenase	41/42	5.36/5.45	0.44	I	U	−2.0
Q72	gi|17987756	BMEI1473	-	3-oxoacyl-(acyl carrier protein) synthase II	44/48	5.36/5.50	0.49	IQ	C	2.3
Translation										
Q8	gi|17987120	BMEI0837	gltX	glutamyl-tRNA synthetase	55/49	5.85/5.66	0.42	J	C	−6.4
Q24	gi|17988198	BMEI1915	rpsA	30S ribosomal protein S1	64/33	5.19/5.67	0.20	J	C	−3.9
Q46	gi|17983840	BMEI1806	trpRS	tryptophanyl-tRNA synthetase	42/41	6.28/6.02	0.27	J	C	−2.6
Q31	gi|17987025	BMEI0742	tuf	elongation factor Tu	43/43	5.29/5.57	0.54	J	C	T
Q64	gi|17988070	BMEI1787	raiA	putative sigma54 modulation protein/SSU ribosomal protein S30P	22/31	5.24/5.50	0.67	J	U	2.9
Transcription										
Q26	gi|17989223	BMEII0878	gntR	transcriptional regulator, GntR family	26/32	6.15/6.61	0.47	K	C	−12.2
Q29	gi|17989461	BMEII1116	vjbR	transcriptional activator, LuxR family	26/32	6.21/6.41	0.80	K	U	−2.0
Cell wall/membrane biogenesis										
Q13	gi|17989189	BMEII0844	omp31	31 kDa outer-membrane immunogenic protein precursor	23/45	5.21/5.08	0.38	M	OM	Y
Q15	gi|17988112	BMEI1829	omp25c	25 KDa outer-membrane immunogenic protein precursor	25/45	4.79/4.57	0.42	M	OM	Y
Q36	gi|17987532	BMEI1249	omp25	25 KDa outer-membrane immunogenic protein precursor	23/30	8.58/5.26	0.31	M	OM	Y
Q37	gi|17988725	BMEII0380	acrA	acriflavin resistance protein A precursor	42/41	5.68/5.51	0.30	M	CM	Y
Q38	gi|17987010	BMEI0727	ddl	D-alanine–D-alanine ligase A	39/43	4.96/5.17	0.41	M	C	Y
Q19	gi|17987532	BMEI1249	omp25	25 KDa outer-membrane immunogenic protein precursor	23/28	8.58/5.07	0.47	M	OM	−34.8
Q17	gi|17988112	BMEI1829	omp25c	25 KDa outer-membrane immunogenic protein precursor	25/31	4.79/4.37	0.42	M	OM	−19.9
Q23	gi|17987532	BMEI1249	omp25	25 KDa outer-membrane immunogenic protein precursor	23/26	8.58/5.67	0.31	M	OM	−19.2
Q22	gi|17987532	BMEI1249	omp25	25 KDa outer-membrane immunogenic protein precursor	23/27	8.58/5.26	0.44	M	OM	−9.6
Q12	gi|17987532	BMEI1249	omp25	25 KDa outer-membrane immunogenic protein precursor	23/41	8.58/5.26	0.50	M	OM	−9.1
Q18	gi|17987532	BMEI1249	omp25	25 KDa outer-membrane immunogenic protein precursor	23/28	8.58/4.88	0.21	M	OM	−8.6
Q16	gi|17987290	BMEI1007	omp25a	25 KDa outer-membrane immunogenic protein precursor	25/34	4.72/4.37	0.42	M	OM	−5.7
Q58	gi|17987532	BMEI1249	omp25	25 KDa outer-membrane immunogenic protein precursor	23/25	8.58/5.68	0.62	M	OM	−3.9
Q11	gi|17989189	BMEII0844	omp31	31 kDa outer-membrane immunogenic protein precursor	23/48	5.21/5.20	0.38	M	OM	−3.1
Q20	gi|17987532	BMEI1249	omp25	25 KDa outer-membrane immunogenic protein precursor	23/28	8.58/4.69	0.41	M	OM	−2.8
Q76	gi|17989189	BMEII0844	omp31	31 kDa outer-membrane immunogenic protein precursor	23/32	5.21/4.88	0.49	M	OM	−2.1
Posttranslational modification, protein turnover, chaperones										
Q14	gi|17988285	BMEI2002	dnaK	molecular chaperone DnaK	69/40	4.86/4.82	0.16	O	C	Y
Q75	gi|17988746	BMEII0401	-	thioredoxin	35/37	4.94/5.03	0.37	O	C	−2.9
Q6	gi|17987613	BMEI1330	htrA	protease DO	53/40	5.81/5.99	0.17	O	U	−2.4
Q68	gi|17989393	BMEII1048	groEL	chaperonin GroEL	57/34	5.04/5.13	0.27	O	C	−2.3
Q40	gi|17987352	BMEI1069	tf	trigger factor	54/58	4.94/5.07	0.55	O	C	−2.0
Inorganic ion transport and metabolism										
Q60	gi|17988349	BMEII0005	modA	molybdate-binding periplasmic protein	25/29	5.31/5.44	0.45	P	P	−2.2
Q32	gi|17988929	BMEII0584	afuA	iron(iii)-binding periplasmic protein precursor	36/38	5.20/5.17	0.49	P	P	−2.0
Q28	gi|17988124	BMEI1841	sbp	sulfate-binding protein precursor	36/41	5.47/6.07	0.34	P	P	4.0
Q69	gi|17986956	BMEI0673	cysP	thiosulfate-binding protein precursor	37/37	5.31/5.23	0.29	P	P	2.3
Q70	gi|17986956	BMEI0673	cysP	thiosulfate-binding protein precursor	37/37	5.31/5.34	0.41	P	P	2.3
Q62	gi|17988952	BMEII0607	fatB	ferric anguibactin-binding protein	31/33	5.36/5.64	0.34	P	P	2.2
General function prediction only										
Q5	gi|17987208	BMEI0925	adh	alcohol dehydrogenase	35/37	5.56/5.99	0.28	R	C	Y
Q21	gi|17986557	BMEI0273	glcG	GlcG protein	14/21	5.10/5.39	0.61	R	U	−5.0
Q48	gi|17989217	BMEII0872	fabG	3-ketoacyl-(acyl-carrier-protein) reductase	28/36	5.57/6.07	0.45	QR	C	−3.0
Q33	gi|17988824	BMEII0479	-	ABC transporter substrate-binding protein	40/40	5.11/5.33	0.39	R	U	3.5
Q52	gi|17987518	BMEI1235	fabG	short-chain dehydrogenase	25/29	6.15/6.79	0.61	QR	C	3.4
Intracellular trafficking and secretion										
Q25	gi|17986405	BMEI0121	secA	protein translocase, chain secA	103/31	5.15/5.66	0.20	U	C	−5.3
Function unknown										
Q59	gi|17986596	BMEI0313	zapA	Hypothetical Cytosolic Protein	13/15	4.78/4.81	0.84	S	C	−2.5
Q42	gi|17987476	BMEI1193	-	cell wall degradation protein	45/43	7.03/6.90	0.45	S	U	2.2
not in COGs										
Q41	gi|17986462	BMEI0178	-	hypothetical protein BMEI0178	18/52	4.79/5.03	0.66	-	U	−5.9
Q56	gi|17986457	BMEI0173	-	YciI-like protein	40/14	5.52/5.74	0.92	-	U	−5.0
Q45	gi|17988858	BMEII0513	gpd	glucose-6-phosphate 1-dehydrogenase	55/31	5.74/6.54	0.28	-	U	−3.6
Q61	gi|17988500	BMEII0156	motD	chemotaxis motd protein	40/31	5.12/5.82	0.17	-	C	−2.5
Q2	gi|17987549	BMEI1266	pdxA	4-hydroxythreonine-4-phosphate dehydrogenase	43/39	9.13/6.63	0.40	-	C	−2.3
Q78	gi|17986825	BMEI0542	-	hypothetical protein BMEI0542	30/30	4.83/4.79	0.64	-	U	−2.2
Q57	gi|17986528	BMEI0244	-	putative translaldolase	23/30	5.69/5.72	0.60	-	U	2.8
Q55	gi|17987937	BMEI1654	-	urease gamma subunit	9/14	5.54/6.49	0.89	-	C	2.1

a Abbreviation of cellular role categories of theoretical (http://www.ncbi.nlm.gov/COG/).

b Abbreviation of cellular location. Protein cellular location was annotated by PSORTb V. 2.0 (http://www.psort.org/). C: Cytoplasmic, P: Periplasmic, U: Unknown, OM: OuterMembrane, CM: CytoplasmicMembrane.

c Proteins upshifted in the BMΔvirB mutant are marked with “+”, and those downshifted with “−”; unique protein spots in BM are marked with “Y”, and in BMΔvirB with “T”.

### virB affect expression of proteins of several function categories

#### Outer membrane proteins

After inactivation of *virB*, the greatest protein expression change was observed in the outer membrane proteins. In the genome of *B. melitensis*, 4 omp25 genes are predicted: BMEI1249 (omp25), BMEI1007 (omp25a), BMEI1829 (omp25c) and BMEI1830 (omp25d). Of the 4 genes, products of 3 of them (BMEI1007, BMEI1249 and BMEI1829) were detected to be differentially expressed. Interestingly, Omp25 and Omp25c were assigned more than one protein spots on the 2-DE profiles. 8 protein spots of BMEI1249 and 2 of BMEI1829 were detected. All these protein spots were down-regulated in BMΔvirB. Omp25 belongs to the OmpA protein family, whose abundance accounts for 30–40% of the outer membrane. The different protein spots might come from post translation modification or breakdown of OmpA proteins, which has been observed in many other bacteria genus. Omp31 is one of the protective antigens of *Brucella*. It is also a hemin binding protein involved in iron uptake. Different products of Omp31 were also observed on 2-DE gel. 3 protein spots of omp31 were all down-regulated in BMΔvirB. Besides omp25 and omp31, some other outer membrane proteins, such as AcrA and Imp, were also down-regulated in *virB* mutant.

#### Heat shock response proteins

To survive in hostile environments, intracellular bacteria induce a number of stress response proteins to adapt to the hostile environments in host cell. In the present study, we found that some heat shock proteins and molecular chaperons were down-expressed in BMΔvirB. These proteins included serine protease HtrA (spot Q6), molecular chaperon DnaK (spot Q14), trigger factor Tf (spot Q40), and chaperonin GroEL (spot Q68). HtrA is generally thought to serve as a stress response protease in the periplasmic space, degrading damaged proteins resulted from a variety of environmental stresses, including elevated temperatures and reactive oxygen intermediates. HtrA is important for adapting to the intracellular environment of host macrophages [17]. Previous studies showed that DnaK was important for *Brucella* growth and survival under stress conditions and macrophage infection. The constitutive *dnaK* mutant failed to multiply in murine macrophage-like cells and was rapidly eliminated in a mouse infection model, implying stress-mediated and heat shock promoter-dependent induction of *dnaK* is a crucial event in the intracellular replication of *B. suis* [18]. Tf is an ATP-independent chaperone and displays chaperone and peptidyl- prolyl-cis-trans-isomerase activities in vitro. In the *E.coli* cytosol, Tf was found to be the first chaperone that binds to the nascent polypeptide chain [19]. Moreover, recently Tf was revealed for the first time as a protective antigen against Brucellosis, implying its important roles [20].

#### Energy production and conversion

A number of proteins associated with energy production and metabolism were also down-expressed in the BMΔvirB. Those proteins including ATP synthase subunit B AtpD (spot Q39 and Q77), alcohol dehydrogenase class III adh (spot Q1), succinate-semialdehyde dehydrogenase PutA (spot Q3), glycerol trinitrate reductase (spot Q47), and succinyl-CoA synthetase subunit beta SucC (spot Q74). AtpD was down-expressed in BMΔvirB, implying ATP synthesis may be reduced, which can influence the energy production. The downshift of acetyl-CoA carboxylase carboxyltransferase subunit alpha AccA (spot Q44) and 3-ketoacyl-(acyl-carrier-protein) reductase FabG (spot Q63) in BMΔvirB indicated down-regulation of fatty acid metabolism. Adipoid, especial phosphatide and cholesterol are the main components of cell membrane, which keep the integrity of the cell membrane. Down-regulation of those proteins may affect the integrity of the cell membrane and may alter resistance of the pathogen against hostile environments in the host. In addition, some proteins associated with amino acid transport and metabolisms were also down-expressed in the BMΔvirB. These include shikimate 5-dehydrogenase AroE (spot Q27), N-acetyl-gamma-glutamyl-phosphate reductase ArgC (spot Q50), and high-affinity branched-chain amino acid transport ATP-binding protein LivF (spot Q51). It is possible that the down-regulation of these proteins might result in decreased levels of ATP.

#### Iron metabolism

In pathogenic bacteria, iron acquisition is critical for the survival in infection. After inactivation of *virB*, expression of some proteins associated with iron metabolism was changed. Ferric anguibactin-binding protein FatB (sport 62) was up- regulated, while iron (iii)-binding periplasmic protein precursor AfuA (sport 32) was down-regulated. AfuA was responsible for junction and transportation of iron. Down-regulated this protein may influence the ability of iron transportation and acquisition of BMΔvirB.

#### Transcription and Translation

GntR (spot Q26) and VjbR (spot Q29) belong to transcriptional regulatory proteins, which involved in virulence gene control in *B. melitensis*. They were down-regulated in BMΔvirB. GntR is a major global regulation protein in *B. melitensis* 16 M. Previous study showed that GntR control *virB* at the transcriptional level. GntR mutant showed decreased survival in cellular models and mice modals. The requirement of a functional *gntR* gene for survival or proliferation in BALB/c mice spleen suggests that the absence of an appropriate control of the Ashwell pathway unsettled the sugar metabolism of the bacteria in mice [21]. VjbR is a member of the family of LuxR, which belong to the quorum sensing system. Previous studies proved that VjbR can regulate the expression of *virB*, which was consistent with our results [22]. Down-regulated of this protein may influence virulence of the mutant. Additionally, some proteins associated to protein synthesis were also down-regulated in BMΔvirB. These proteins included glutamyl-tRNA synthetase GltX (spot Q8), tryptophanyl tRNA synthetase TrpRS (spot Q46) and 30S ribosomal protein S1 RpsA(spot Q24). Down regulation of these proteins might result in decreased synthesis of some related proteins.

### VirB affects transcription of dnaK, vjbR, omp25, htrA and gntR during macrophage cell infection

Among the identified proteins, several known virulence related proteins, including VjbR, HtrA, GntR, Omp25 and DnaK, were down-regulated when *virB* was inactivatived (Figure 4A). Simi-quantitative RT-PCR of these genes showed that transcription of them was decreased in the *virB* mutant (Figure 4B), being consistent with results from comparative proteomes, indicating that these genes were affected by the *virB*.

Because *virB* is primarily activated during infection, we attempted to test whether these virulent genes were affected by the *virB* during macrophage cell infection. Firstly, quantitative real time PCR method was developed for these genes. Total RNA was isolated from infection mixtures of macrophages and bacteria followed by DNase I treatment and reverse transcription. Coding region of *omp25* could be amplified from cDNA and DNA, but not RNA, indicating that the RNA was not contaminated by DNA (data not shown). By using cDNA from infection mixtures or uninfected macrophage as template, *virB*, *dnaK*, *htrA*, *omp25* and *gntR* was amplified with SYBR Green I method. All of them showed a unique Tm for infection mixture but not for uninfected macrophage, indicating that the amplification is specific (data not shown). Amplification efficiencies of these genes are all close to 2. Taken together, the real time PCR could be used for relative transcription quantification.

**Figure 5:**
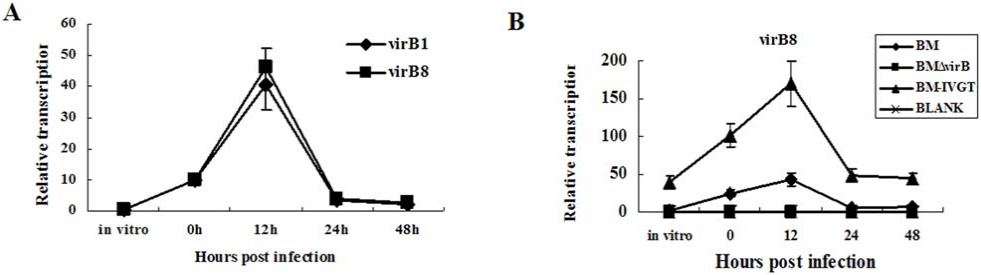
Transcriptional profile of *virB* during host cell infection. Macrophage like cell J774A.1 were infected with BM, BMΔvirB and BM-IVGT. At different time (0, 12, 24 and 48 h) post the infection, RNA was isolated from the infection mixtures and reverse transcribed into cDNA. Transcription of *virB* genes were then quantified by qRT-PCR. A: Relative transcription of *virB1* and *virB8*. The *virB1* and *virB8* was identically transcribed. B: Relative transcription of *virB8* in BM, BMΔvirB and BM-IVGT. Transcription of *virB8* peaked at 12 h and then decreased in BM and BM-IVGT. No transcription of *virB8* was detected in BMΔvirB and uninfected macrophage cells.

Then we analyzed the transcription of *virB* genes during host cell infection. To obviate the possibility of transcription preference, *virB1* and *virB8*, two genes located at different locus of the *virB* operon, were selected. The two genes showed identical transcription profiles, confirming that the *virB* is an operon, and any one of its genes can represent its transcription (Figure 5A). As shown in figure 5B, immediately after enter macrophage, the *virB* was activated, and the transcription peaked at 12 h post the infection and then decreased.

**Figure 6:**
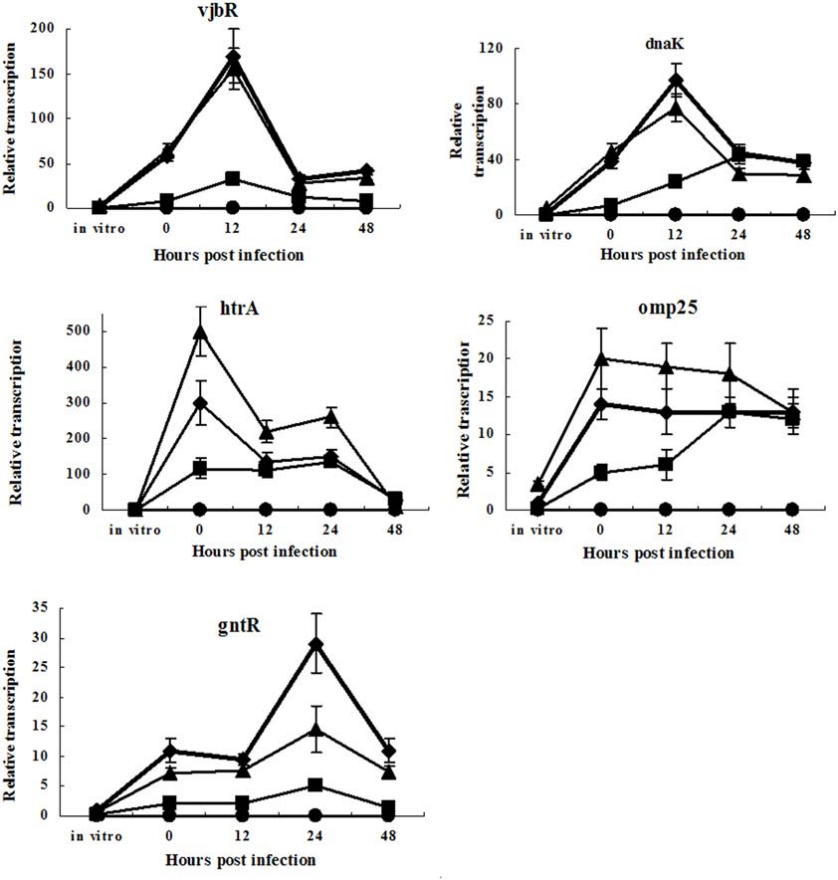
Transcription of *vjbR*, *dnaK*, *htrA*, *omp25*, and *gntR* during host cell infection (⧫— BM; ▪ — BMΔvirB; ▴ — BM-IVGT; •—BLANK). Macrophage cells were infected with BM, BMΔvirB and BM-IVGT. At different time (0, 12, 24 and 48 h) post the infection, RNA was isolated from the infection mixtures and reverse transcribed into cDNA. Transcription of selected genes was then quantified by qRT-PCR.

Then, transcription of *dnaK*, *vjbR*, *omp25* (BMEI1249), *htrA* and *gntR* in BM, BMΔvirB and BM-IVGT were quantified and compared. For BM, *dnaK*, *vjbR*, *omp25*, *htrA* and *gntR* were transcribed at a very low level in vitro (TSB 7.0), but were highly activated immediately in macrophage, indicating these genes play important role in *Brucella* intracellular survival. Transcription of *htrA* peaked at 0 h, *vjbR* and *dnaK* at 12 h, and *gntR* at 24 h, implying that they function at different stage of infection (Figure 6). The transcription of these genes were greatly decreased in BMΔvirB, but were recovered to some extent in BM-IVGT, indicating that transcriptions of these genes are regulated by the *virB* operon in a positive manner.

### virB positively regulates vjbR in a cell density–dependent manner

**Figure 7:**
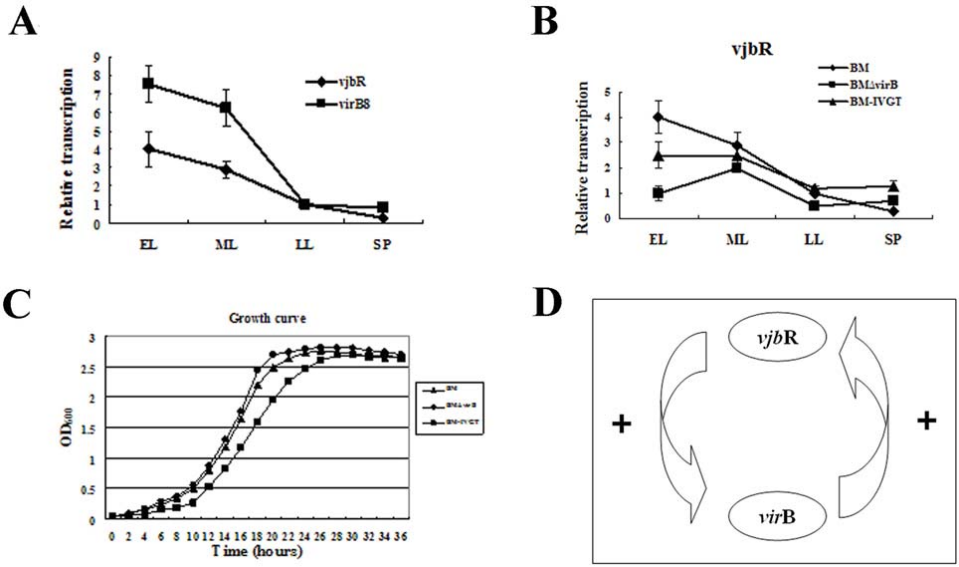
*vir*B positively regulates *vjb*R. A) Transcription of *vjb*R and *vir*B8 during the early logarithmic (EL), mid-logarithmic (ML), late logarithmic (LL), and stationary phases (SP) demonstrate that *vjb*R and *vir*B8 are greatly transcribed in EL and ML. B) Transcription of *vjb*R in BM, BMΔvirB, and BM-IVGT at EL, ML, LL, and SP show that v*jb*R is down-regulated at EL in BMΔvirB. C) BMΔvirB demonstrated higher growth rate than BM and BM-IVGT. D) *vir*B and *vjb*R positively regulate each other and form a positive regulation circuit.

Transcription analyses of in vitro stress, macrophage infection, and mouse models indicated that *vir*B positively regulates *vjb*R, a quorum sensing (QS) regulator that functions in a cell-dependent manner. To test whether the *vir*B regulated *vjb*R in a cell density–dependent manner, the transcription of *vir*B and *vjb*R at different growth phases were compared. *vir*B and *vjb*R had similar transcription profiles (Figure 7A). Their transcription peaked during the early logarithmic phase and was down-regulated with cell density increase, indicating their growth phase–dependent transcription.

We then compared the transcription of *vjb*R in BM, BMΔvirB, and BM-IVGT at different growth phases. Compared to BM, transcription of *vjb*R was decreased in BMΔvirB and recovered in BM-IVGT. Transcription of *vjb*R in BMΔvirB peaked at the mid-logarithmic phase, in contrast to BM and BM-IVGT (Figure 7B). *vjb*R seemed to inhibit growth of *Brucella* to some extent [22]. To test whether BMΔvirB has growth defects, the growth rates of the 3 bacteria were compared. BMΔvirB had a higher growth rate than BM (Figure 7C). In the BM-IVGT mutant, where *vir*B is highly transcribed, the growth rate was inhibited, implying that *vir*B has inhibitory effects on growth of *Brucella* (Figure 7C).

Based on these observations, it can be concluded that *vjb*R is positively regulated by *vir*B. On the other hand, the *vjb*R mutant has down-regulated expression of *vir*B, indicating that *vir*B is also positive regulated by *vjb*R [22]. Therefore, *vir*B and *vjb*R positively regulate one another, forming a positive regulation circuit (Figure 7D).

## Discussion

Pathogen-host interactions during bacterial infection expose bacteria to multiple physiological and biological stresses, and intracellular pathogens are known to adapt to changes in their environment, avoiding degradation by host cell defense systems by coordinated regulation of gene expression. The *virB* operon is essential for intracellular survival and chronic infection of several *Brucella* species [8], and previous studies implied that this operon regulates virulence genes that are also important for *Brucella* survival [4], [10]. So far, many studies proved that T4SS is essential for bacterial survival, but there have no reports on the proteins that affected by the *virB* operon. In the present study, comparative proteome approach was used to define the potential target proteins affected by *virB*.

A number of heat shock proteins and molecular chaperons were down-shifted in BMΔvirB. These proteins play an essential role as part of protein repair systems in protecting the bacteria under the environment encountered in the phagosome, and degrading damaged proteins resulting from exposure to a variety of environmental stresses, including elevated temperatures and exposure to reactive oxygen intermediates. At the same time, these proteins are involved in folding and proper localization of virulence factors. DnaK is a heat shock protein previously shown to be important for bacterial growth and survival under conditions of high temperature, low pH, oxidative stress, and macrophage infection [18], [23]. It is speculated that *dnaK* induction, possibly through low pH or starvation, is required for correct synthesis of certain virulence proteins that mediate *Brucella* survival during stress [23]. DnaK activation was partially dependent on *virB*, as its induction was inhibited in BMΔvirB. Bacterial stress response proteins of the high temperature requirement A (HtrA) family are serine proteases which appear to play an important role in scavenging oxidatively damaged proteins from the cell before they reach toxic levels [24], [25], [26]. The necessity for HtrA in virulence is believed to be related to its ability to protect cells from the products of the oxidative burst of host macrophages. *Brucella htrA* mutants have been described as being temperature sensitive, sensitive to oxidative killing in vitro, sensitive to killing by macrophages, and attenuated in both mice and ruminants [17], [27], [28]. These characteristics are consistent with the proposed function of the HtrA protease and similar to those described for *htrA* mutants of *Escherichia coli*, *Yersinia enterocolitica*, and *Legionella pneumophila* [25], [29], [30]. Down-regulation of these heat shock proteins and molecular chaperones in BMΔvirB implied that *virB* might affect *Brucella* adaptation to in vitro and in vivo environments.

Intracellular proteolytic degradation is important in bacteria for the elimination of damaged proteins, modulation of protein levels, and maintenance of amino acid pools. Proteolytic enzymes represent one of the best investigated classes of proteins. For many years it was accepted that proteolytic enzymes functioned primarily in the acquisition of nutrients for growth and proliferation through the degradation of host tissues. However, recent observations indicated that pathogen-derived proteolytic enzymes also play important roles in the regulation of critical host processes, which is critical for survival of the invading microbes in a hostile host environment [31]. Proteins involved in proteolytic degradation, including heat shock protein, molecular chaperon and proteolytic enzymes are crucial for the intracellular survival of *Brucella*. Two proteolytic enzymes were down-expressed in BMΔvirB. This indicated that the *virB* operon is also needed to eliminate damaged proteins for *Brucella*.

Bacteria have the capability to express an appropriate subset of genes conferring a growth or survival advantage in a given situation. The expression of bacterial genes is regulated at the initiation of transcription by regulators which, in response to specific environmental and/or cellular signals, bind at the promoter of target genes to activate or repress them. Several important virulence related proteins involved in intracellular survival, including VjbR, DnaK, HtrA, Omp25, and GntR, were down-regulated in BMΔvirB. Quantitative RT-PCR showed that transcription of these virulence related genes was also affected by *virB* during macrophage cell infection. This was consistent with an observed decreased survival of the *virB* mutant in macrophage (data not shown), indicating that *virB* may mediate *Brucella* intracellular survival by affecting the expression of virulence related genes.

Intracellular pathogens are known to adapt to changes in their environments in order to survive under the stresses encountered in host cells [12]. Acidic intraphagosomal pH developed from proton pump activation [32], and bacteria are generally sensitive to low pH. However, *Brucella* can survive at pH 4. Indeed, studies suggest that early (but not late) acidification of BCVs may lead to gene expression required for *Brucella* to adapt to and survive in its host [33]. *VirB* is activated in the early stages of host cell infection, and blocking early acidification inhibits both expression of *virB* and *Brucella* survival [9]. Our results indicated that *virB* and the virulence related genes affected by the *virB* operon were also activated during early infection. Thus, *Brucella* may adapt to its intracellular environment and avoid degradation by the coordinated regulation of gene expression in the early phase of infection.

Type IV secretion machinery is a membrane associated structure. Disruption of this structure seemed to result in great modifications in membrane and other components. Omp25 is a trans-membrane protein that is present on the outer membrane of *Brucella* and is probably covalently bound to the underlying peptidoglycan layer of the cell [34]. Regarding virulence, mutant of *B. melitensis*, *B. abortus*, and *B. ovis* strains with the *omp25* gene inactivated have been found to be attenuated in mice, goats, and cattle [35], [36], [37]. Additionally, Omp25 is also an important immune response regulator, and may mediate inhibition the host cell production of TNF-α [38]. Indeed, down-regulation of Omp25 and subsequent production of TNF-α may be one reason for the high clearance of the *virB* mutant from its host. Iron acquisition is critical for the survival of pathogenic bacteria during infection. Successful pathogenic bacteria have evolved a variety of strategies in order to acquire iron from the iron or haem resources of mammalian hosts. In our study we found that iron metabolism was changed in BMΔvirB. Previous study proved that haem may be a relevant iron source for *Brucella* during intracellular replication. Outer membrane protein Omp31 present in most *Brucella* species is a haemin-binding protein. Down-expressed of Omp31 may reduce the iron acquisition in BMΔvirB. We presumed that the altered iron metabolism and down-expressed of Omp31 may influence the iron acquisition, and therefore affected the bacteria survival during intracellular infection.

Two QS genes have been predicted in *Brucella*, one of which (*vjbR*) is involved in *Brucella* virulence [9], [39]. The transcription analysis during macrophage infection indicates that *virB* positively regulates the expression of *vjbR*. Previous studies demonstrate that *virB* is also activated by *vjbR* [22]. It is possible that *virB* and *vjbR* may regulate each other, forming a regulation circuit. As *virB* encodes type IV secrection machinery, it may be that the effector proteins of *virB* are responsible for the directly regulation of *vjb*R. Therefore, identification of these effector proteins may provide insight into this regulation circuit.

The key function of T4SS of *Brucella* is to direct intracellular trafficking of BCV to reach replicative niche in the ER. And during this process, the effector proteins may play essential roles. A recent study showed that two proteins, VceA and VceC, were translocated by T4SS into macrophage[40]. It is possible that the two effectors, and other unidentified effector proteins are involved in the *vir*B mediated *Brucella* intracellular survival. In the present study, by comparing the proteome of wild type strain and that of a *vir*B mutant under identical in vitro stress condition, a number of proteins of several categories were found to be differentially expressed. Because the in vitro condition is a stress condition that used to simulate intracellular environment, the differentially expressed proteins identified are mainly the ones involved in stress resistance mechanisms. Most of these proteins are possibly involved in *Brucella* adaptation to intracellular environments. The differential expression of stress response proteins seemed not to be contrast to the essential role of virB in BCV intracellular trafficking. During the intracellular process, it is very possible that the *vir*B also affects expression of other proteins. These proteins identified are mainly those affected indirectly by virB. Take into account previous study and results from our present study, it is possible that, beside secret effector proteins into host cells for intracellular survival, as a membrane structure, T4SS also affect expression of genes involved in adaptation to intracellular environments.

### Conclusions

The analysis of a *virB* mutant using comparative proteomics and qRT-PCR defined the target proteins affected by the *virB* operon. The inactivation of the *virB* operon affected a series of known and unknown proteins and many of these differentially expressed proteins are involved in the adaptation of *Brucella* to intracellular environments. Therefore, in addition to the most important roles in intracellular trafficking of BCV, the *virB* operon may contribute the adaptation of *Brucella* to hostile environments and survival in the host cell by affecting the expression of proteins associated with the intracellular survival.
